# Discovery of non-climacteric and suppressed climacteric bud sport mutations originating from a climacteric Japanese plum cultivar (*Prunus salicina* Lindl.)

**DOI:** 10.3389/fpls.2015.00316

**Published:** 2015-05-12

**Authors:** Ioannis S. Minas, Carolina Font i Forcada, Gerald S. Dangl, Thomas M. Gradziel, Abhaya M. Dandekar, Carlos H. Crisosto

**Affiliations:** ^1^Department of Plant Sciences, University of California, DavisDavis, CA, USA; ^2^Foundation Plant Services, University of California, DavisDavis CA, USA

**Keywords:** ethylene, genetic analysis, microsatellite markers, propylene, ripening, softening, 1-methylcyclopropene

## Abstract

Japanese plums are classified as climacteric; however, some economically important cultivars selected in California produce very little ethylene and require long ripening both “on” and “off” the tree to reach eating-ripe firmness. To unravel the ripening behavior of different Japanese plum cultivars, ripening was examined in the absence (air) or in the presence of ethylene or propylene (an ethylene analog) following a treatment or not with 1-methylcyclopropene (1-MCP, an ethylene action inhibitor). Detailed physiological studies revealed for the first time three distinct ripening types in plum fruit: climacteric, suppressed-climacteric, and non-climacteric. Responding to exogenous ethylene or propylene, the slow-softening supressed-climacteric cultivars produced detectable amounts of ethylene, in contrast to the novel non-climacteric cultivar that produced no ethylene and softened extremely slowly. Genetic analysis using microsatellite markers produced identical DNA profiles for the climacteric cultivars “Santa Rosa” and “July Santa Rosa,” the suppressed-climacteric cultivars “Late Santa Rosa,” “Casselman,” and “Roysum” and the novel non-climacteric “Sweet Miriam,” as expected since historic records present most of these cultivars as bud-sport mutations derived initially from “Santa Rosa.” This present study provides a novel fruit system to address the molecular basis of ripening and to develop markers that assist breeders in providing high-quality stone fruit cultivars that can remain “on-tree,” increasing fruit flavor, saving harvesting costs, and potentially reducing the need for low-temperature storage during postharvest handling.

## Introduction

Fleshy fruit ripening is a genetically regulated process that coincides with seed maturation, advancing many physicochemical changes that transform a fertilized ovary into a tasty, nutritious, and appealing fruit that attracts animals and promotes dispersal of mature seeds (Giovannoni, 2004). Over-ripening, rapid softening, and susceptibility to chilling injury are limiting factors of plum postharvest life and fruit consumption (Crisosto et al., 2004; Crisosto and Day, 2011). Precise understanding of the processes underlying plum fruit ripening is key to managing softening during maturation-ripening (“on-tree”) and postharvest handling (“off-tree”) to increase storage potential, protect consumer quality and reduce postharvest losses. Fruits are classified as climacteric or non-climacteric according to their ripening behavior (Burg and Burg, 1965; Giovannoni, 2001). During postharvest ripening, physiologically mature climacteric fruits exhibit a logarithmic (autocatalytic) increase in endogenous ethylene biosynthesis (system II) and respiration (Lelievre et al., 1997; Giovannoni, 2004). It is generally accepted that climacteric fruit cell wall remodeling and softening is triggered by endogenous or exogenous ethylene, which also mediates expression of specific genes and transcription factors involved in primary (soluble sugars, organic acids) and secondary (aroma volatiles and anthocyanin biosynthesis) metabolism and defines fruit quality development (Osorio et al., 2013; Tohge et al., 2014). In contrast, most non-climacteric fruits can fully ripen only if they are allowed to remain attached to the parent plant; if they are detached, they produce only basal concentrations of ethylene (system I), while respiration decreases during maintenance at room temperature (Lelievre et al., 1997). Exposure of fruit to exogenous propylene (an analog of ethylene) can induce autocatalytic endogenous ethylene production in climacteric fruit only and serves as a second criterion to distinguish climacteric and non-climacteric fruits (Burg and Burg, 1965).

Japanese plum (*Prunus salicina* Lindl.) is a diploid fruit tree (2*n* = 2 × = 16) of the *Rosaceae* family that has been classified historically as climacteric, with ethylene controlling changes during ripening. Once synthesized, ethylene interacts with a family of membrane-bound receptors such as ethylene receptor (ETR) and ethylene response sensor (ERS) that in the absence of the hormone, actively suppress ethylene responses (Klee and Giovannoni, 2011). Upon ethylene binding, the response's suppression is removed. The signal is transmitted into the nucleus and consequently amplified by a transcription factor cascade, which includes ethylene-insensitive (EIN) and EIN-like-proteins (EILs) (Solano et al., 1998; Klee and Giovannoni, 2011). Finally, members of the APETALA2/ethylene responsive factor (AP2/ERF) transcription factor family, which include ERFs, are involved in a feedback loop that stimulates autocatalytic ethylene synthesis and binds *cis*-elements found in the promoters of target genes, modulating their transcription and thereby inducing downstream ethylene responses that lead to fruit ripening (Wang et al., 2002; Grierson, 2013). Interestingly, some plum cultivars display a suppressed-climacteric ripening pattern characterized by a slow softening/ripening profile due to reduced capacity to convert 1-amino-cyclopropane-1-carboxylic acid (ACC) to ethylene (Abdi et al., 1997, 1998). Recent studies reported differences in the mRNA accumulation patterns of four ethylene perception and signal transduction components: *ETR1, ERS1, CTR1* (constitutive triple-response protein kinase), and *ERF1* (El-Sharkawy et al., 2007), four members of the ACC-synthase gene family (*ACS1, ACS3a, ACS3b, ACS4*) (El-Sharkawy et al., 2008), and auxin-mediated control of ethylene-responsive transcriptional factors (*ERFs*) and germin-like proteins (GLPs) (El-Sharkawy et al., 2009, 2010) among suppressed-climacteric and climacteric plum cultivars. When these suppressed-climacteric cultivars are exposed to ethylene or propylene, autocatalytic ethylene production is slowly induced, but their climacteric peak is half that of typical climacteric cultivars, so they should still be classified as climacteric (Abdi et al., 1997, 1998).

The previous two decades of research on the ripening biology of climacteric and non-climacteric fruits have mainly used tomato as the model climacteric fruit and strawberry as the model non-climacteric fruit (Osorio et al., 2013). Since is difficult to conduct comparative molecular studies on climacteric and non-climacteric fruit originating from different species, fundamental questions about fruit ripening biology are preferentially addressed using ripening mutants or transgenic lines (Giovannoni, 2007). Fruit-type mutants and transgenic lines can facilitate molecular analysis because they are genetically similar to their wild-types (wt) and the resulting associations provide a common basis for studies with fleshy fruits. The discovery and/or creation of pleiotropic ripening mutations of tomato such as ripening-inhibitor (rin), non-ripening (nor), Never-ripe (Nr), Colorless non-ripening (Cnr), Green-ripe (Gr) (Herner and Sink, 1973; McGlasson et al., 1975; Giovannoni, 2007, 2001), anti-sense tomato (Oeller et al., 1991; Picton et al., 1993) and melon (Ayub et al., 1996) fruit shed light on the active role of ethylene in fruit ripening. In contrast, no significant molecular studies in the field of ripening biology have been conducted in perennial plants such as fruit trees, except for some attempts using ethylene-silenced transgenic apples (Dandekar et al., 2004) and kiwifruit knockdown lines (Atkinson et al., 2011), due to the difficulty of creating such mutants or transgenic lines. Marketing of such mutant and/or genetically modified (GMO) fruit has been limited by the cost of regulatory compliance, real or perceived consumer concerns and the unacceptable quality of such fruit.

1-Methylcyclopropene (1-MCP), an ethylene action inhibitor (Sisler and Blankenship, 1996) commercialized and registered as SmartFresh (AgroFresh Inc., Rohm and Haas, Spring House, PA, USA) is being used as an alternative to ripening mutants to inhibit ripening and softening and to address the role of ethylene in climacteric and non-climacteric fruit ripening (Watkins, 2006). In plums, inhibition of ethylene production by 1-MCP delayed ripening and softening (Abdi et al., 1998; Martinez-Romero et al., 2003; Minas et al., 2013), making it a candidate for a sustainable postharvest strategy of storing, transporting and retail handling of fruit at higher than normal storage temperatures to avoid chilling injury (CI), protecting flavor while providing energy and cost savings (Minas et al., 2013).

During over two decades of research on plum cultivars with different softening rates, we looked carefully into softening patterns among California cultivars as a natural, sustainable approach to control ripening during maturation/ripening “on-” and “off-tree.” Our hypothesis is that there is a slow-ripening group of commercial, genetically-related plum cultivars, with a flesh firmness loss less than two Newtons per day, some of which behave as suppressed-climacteric (producing reduced ethylene levels in response to exogenous ethylene exposure), and others as non-climacteric (unable to produce any ethylene), in which the role of ethylene in softening regulation and other ripening quality traits should be documented. The aim of our study was to fully characterize the ripening behavior and the softening regulation of the different plum types among this group of cultivars and to identify their genetic relationships to support the development of novel markers for stone fruit-breeding programs and potential practical orchard manipulations. The incorporation of a non-climacteric, slow-softening trait into existing plum breeding programs should lead to the selection of high-quality, sustainable, GMO-free cultivars that can remain “on-tree” longer to increase quality, reduce harvesting costs, and potentially reduce the need for cold storage during postharvest handling.

## Materials and methods

### Fruit material and experimental design

Over three growing seasons, quality measurements were obtained from 13 Japanese plum (*Prunus salicina* Lindl.) cultivars (Table 1) grown in commercial orchards located in the Reedley-Kingsburg, CA, area and in the “Heirloom” plot at the University of California's Kearney Agricultural Research and Extension Center (KARE) in Parlier, CA. Plums of uniform size, free from visual blemishes and diseases, were harvested at the California “well-mature” pre-climacteric stage according to the California Tree Fruit Agreement (Crisosto, 1994) from three randomly selected trees (each tree represented a biological replication), packed in cardboard boxes, and taken within a few hours to the F. Gordon Mitchell Postharvest Laboratory at the KARE Center. Immediately upon arrival, three biological replications of 10 fruits (the “fruit sample”) from each cultivar were used to analyze fruit quality at harvest (H) by measuring fruit color, flesh firmness, soluble solids concentration (SSC) and titratable acidity (TA) as described previously (Minas et al., 2013). In addition to harvest quality measurements, postharvest ripening-softening behavior at 20°C was studied in three independent experiments corresponding to three growing seasons. As a final approach, 43 plum cultivars (Table 2, Okie and Ramming, 1999), including the 13 cultivars characterized in this work, were genetically characterized using 10 microsatellite markers to reveal any relationships among cultivars with distinct ripening behavior.

**Table 1:** **Plum cultivar harvest quality traits**.

**Cultivar**	**Season 1**					**Season 2**				
	**Harvest date**	**Firmness (*N*)^a^**	**SSC^b^ (%)**	**TA^c^ (%)**	**SR^d^**	**Harvest date**	**Firmness (*N*)**	**SSC (%)**	**TA (%)**	**SR**
Ambra	02-Jul	27.1	12.7	1.4	3.7	06-Jul	28.6	12.5	1.6	3.7
Casselman	12-Aug	27.3	15.8	0.8	0.5	10-Aug	35.0	15.6	0.8	0.7
Durado	24-May	28.8	11.9	0.7	2.9	27-May	32.1	11.4	0.8	3.6
Eldorado	14-Jul	27.5	12.0	0.3	2.1	13-Jul	27.1	12.7	0.4	2.7
Friar	28-Jul	28.1	11.1	0.6	3.2	27-Jul	28.1	9.1	0.5	3.0
July Santa Rosa	29-Jun	27.2	11.5	0.9	2.7	05-Jul	27.4	11.7	0.8	2.7
Laroda	14-Jul	34.8	12.1	1.0	1.8	20-Jul	29.4	12.2	1.1	1.2
Late Santa Rosa	14-Jul	34.1	12.1	1.1	1.3	27-Jul	29.2	12.1	1.0	1.0
Roysum	29-Sep	28.2	16.0	0.5	0.6	29-Sep	31.3	13.5	0.4	0.4
Santa Rosa	17-Jun	28.5	12.0	1.1	2.6	21-Jun	30.2	11.2	1.4	2.4
Joanna Red	17-Aug	30.5	16.1	0.6	2.6	16-Aug	29.1	15.8	0.7	2.7
Angeleno	08-Sep	30.3	16.9	0.6	0.5	06-Sep	31.2	16.6	0.8	0.6
Sweet Miriam	12-Oct	29.7	19.7	0.4	0.4	08-Oct	28.4	19.5	0.4	0.2
LSD^e^		3.4	1.1	0.1	0.2		3.1	0.9	0.1	0.2

*Harvest date, flesh firmness, SSC, TA, and SR during postharvest ripening in two seasons of 13 plum cultivars growing at the Kearney Agricultural Research and Extension Center's Heirloom plot in Parlier, CA*.

a *Values represent the mean of three biological replications of ten fruit*.

b *Soluble solids concentration (%)*.

c *Titratable acidity expressed in malic acid*.

d *Softening rate (firmness N loss per day)*.

e *Least significant difference (P = 0.05)*.

**Table 2:** **List of cultivars used for genetic analysis**.

**Cultivar**	**Origin/originator**	**Species (Pedigree)**
Ambra		*P. salicina*
Angeleno	Garabadien (CA)	*P. salicina* (op^a^ Queen Ann)
Autumn Rosa		*P. salicina*
Blackamber	USDA (CA)	*P. salicina* (Friar × Queen Rosa)
Beauty	Burbank	*P. salicina* (Unknown complex hybrid produced by Luther Burbank)
Burbank	Burbank	*P. salicina* (Unknown complex hybrid produced by Luther Burbank)
Burgundy	Dinuba (CA)	*P. salicina* [op (Mariposa × Eldorado)]
Casselman	Casselman (CA)	*P. salicina* (Bud mutation of Late Santa Rosa)
Catalina	Krause (CA)	*P. salicina* (op Angeleno)
Dolly	Modesto (CA)	*P. salicina* (op Red Beaut)
Durado	Fresno (CA)	*P. salicina* (7a-31M × Burmosa)
Eldorado	Burbank	*P. salicina* (Unknown complex hybrid produced by Luther Burbank)
Elephant Heart	Burbank	*P. salicina* (Unknown)
Flavor Queen	Zaiger (CA)	*Prunus* hybrid [Mariposa × (Red Beaut x cot)]
Flavor Supreme	Zaiger (CA)	*Prunus* hybrid (Plum–apricot hybrid)
Fortune	USDA (CA)	*P. salicina* [Laroda × (Queen Ann × Late Santa Rosa)]
Friar	USDA (CA)	*P. salicina* (Gaviota × Nubiana)
Grand Rosa	Anderson (CA)	*P. salicina* (op Eldorado)
Green Gage		*P. domestica*
Joanna Red	Zaiger (CA)	*P. salicina* (Unknown)
July Santa Rosa	Friesen (CA)	*P. salicina* (Bud mutation of Late Santa Rosa)
Kelsey	Japan	*P. salicina* (Cultivar introduced to the US by Luther Burbank)
Laroda	UCD (CA)	*P. salicina* (Gaviota × Santa Rosa)
Late Santa Rosa		*P. salicina*
Marianna 2624	UCD (CA)	*Prunus* hybrid (op Marianna rootstock = *P. cerasifera* × P. *munsoniana*)
Mariposa	Pasadena (CA)	*P. salicina* (Unknown)
Methley	South Africa	*Prunus* hybrid (*P. salicina* × *P. cerasifera*)
Myrobalan A		*P. cerasifera* (op)
Myrobalan B	France	*P. cerasifera* (op)
Myrobalan 29C	Marysville (CA)	*P. cerasifera* (op)
Nubiana	Winters (CA)	*P. salicina* (Gaviota × Eldorado)
Owen T	USDA (CA)	*P. salicina*
Queen Ann	Winters (CA)	*P. salicina* (Gaviota × Eldorado)
Royal Diamond	Kitahara (CA)	*P. salicina* (Unknown)
Roysum	Sumruld (CA)	*P. salicina* (Bud mutation of Late Santa Rosa)
Santa Rosa	Burbank	*P. salicina* (Unknown complex hybrid produced by Luther Burbank)
Satsuma	Visalia (CA)	*P. salicina* (Unknown complex hybrid produced by Luther Burbank)
Shiro	Burbank	*P. salicina* (Unknown complex hybrid produced by Luther Burbank)
St. Julien		*P. salicina* (Unknown former *P. insititia* subspecies of *P. domestica*)
Stanley	Geneva (NY)	*P. domestica* (Agen × Grand Duke)
Sweet Miriam	Fresno (CA)	*P. salicina* (Bud mutation of Santa Rosa)
Sutter	Davis (CA)	*P. domestica* (Unknown)
Wickson	Burbank	*P. salicina* (Unknown complex hybrid produced by Luther Burbank)

*Cultivar name, origin, and pedigree of the 43 plum cultivars studied*.

a *Open pollination*.

### Experiment 1: softening segregation

To segregate the 13 plum cultivars based on their softening patterns, plums immediately after harvest (H) were placed in ventilated jars at 20°C (90% relative humidity, RH) attached to a flow-through system to retain stable flow rates of atmospheric saturated air filtered through potassium permanganate (KMnO_4_, an ethylene oxidizer) at the desired levels using a gas mixing board and micrometering valves (Gas Mixing System, Postharvest Research, Davis, CA, USA) and ripened for up to 10 days (d). Flow rates were adjusted using a digital mass flow meter (model RO-28, Tylan General, Mykrolis Corp., Billerica, MA, USA) to ensure that carbon dioxide (CO_2_) accumulation remained below 0.3% throughout ripening to avoid any interaction with endogenous ethylene biosynthesis (Crisosto et al., 1993). A fruit sample of each cultivar was assessed for flesh firmness at the beginning of ripening (H) and up to 10 d during ripening at 20°C or until fruit were fully ripe (“ready-to-eat” stage), defined as when firmness was equal to or below 10 N. Softening rate was calculated as loss of flesh firmness per day during ripening until fruit flesh firmness reached ≤ 10 N (Crisosto and Day, 2011). Statistical analysis used SPSS 19.0 for Mac OS X (SPSS, Chicago, IL, USA). Data (means of three biological replications) were subjected to analysis of variance and least significant differences (LSD) at the 5% level for means comparison. Graphs were created using Prism v5.0 for Mac OS X (Graph Pad Inc., San Diego, CA, USA).

### Experiment 2: response to exogenous ethylene

To characterize the softening response to exogenous ethylene of all 13 cultivars studied in the previous experiment, fruits from each cultivar were ripened (20°C) under ethylene-free air or under continuous ethylene (10 μL L^−1^) to evaluate their softening patterns. As in the previous season, all plum fruit samples were transferred immediately after harvest to ventilated jars at 20°C to ripen as previously described. In this second season, jars were connected to a flow-through system either ventilated continuously with humidified, ethylene-free air or with humidified air containing exogenous 10 μL L^−1^ ethylene for up to 10 d. Softening rate was calculated in a fruit sample per cultivar as in Experiment 1 (Section Experiment 1: Softening Segregation).

The plum cultivars were segregated based on fruit softening patterns and the slow-softening plums (flesh firmness loss < 2 N per day) were examined further to determine their ability of respond to exogenous ethylene and the possibility that ripening could be accelerated in this type of plum by a short ethylene treatment. Thus, an additional experiment was set up (“intermittent test”). “Roysum” plums immediately after harvest were transferred to 20°C to ripen under air or treated with 10 μL L^−1^ exogenous ethylene for 1, 2, 3, or 4 d using the flow-through system described above. Flesh firmness was determined daily in a fruit sample per ethylene exposure treatment until plums reached the “ready-to-eat” stage (flesh firmness ≤ 10 N) or up to 9 d. Statistical analysis was performed as described in Experiment 1 (Section Experiment 1: Softening Segregation).

### Experiment 3: physiological characterization of the distinct ripening patterns

Based on the results of the previous experiments, response to exogenous ethylene and industry experience, three Japanese plum cultivars with similar harvest dates were selected for their different softening patterns. Over the third growing season a standard recognized climacteric cultivar (“Joanna Red”) and two slow-softening cultivars (“Angeleno” and “Sweet Miriam”) were harvested (Supplementary Table 1), randomized, and subjected to two postharvest treatments: (1) untreated (control, C); and (2) treated with 0.5 μL L^−1^ 1-MCP at 20°C for 24 h (1-MCP treatment, M), as described previously (Minas et al., 2013). Immediately after treatment, fruit samples transferred to room temperature (20°C, 90% RH) to ripen after harvest. During ripening at 20°C, control and 1-MCP-treated fruit samples were split into two ripening treatments: ventilated continuously (1) with humidified, ethylene-free air at a flow rate of 2 L min^−1^ in sealed, 330-L aluminum tanks connected to a flow-through system or (2) with humidified, ethylene-free air containing 500 μL L^−1^ propylene, an analog of ethylene, at the same flow rate. Air streams with or without propylene were prepared in the desired proportions by mixing metered flows of atmospheric air filtered through potassium permanganate (KMnO_4_) and a purchased mixture of 10% (v/v) propylene in N_2_ (Praxair Inc., Danbury, CT, USA) using a gas mixing board and micrometering valves (Gas Mixing System, Postharvest Research, Davis, CA, USA), then the gas mixture was bubbled through distilled water to maintain ~ 90% RH. This produced four postharvest treatments: (1) control fruit ripened in air (control-air, C-A); (2) control fruit ripened in propylene (control-propylene, C-P), (3) 1-MCP-treated fruit ripened in air (1-MCP-air, M-A) and (4) 1-MCP-treated fruit ripened in propylene (1-MCP-propylene, M-P).

Ethylene, carbon dioxide (CO_2_) and propylene concentrations were monitored daily in the 330-L tanks and in five 0.7-L jars per gas combination. These jars contained one fruit each and connected to the same flow-through system, ventilated continuously with humidified air with or without propylene. Ethylene production and respiration rate of the fruit was analyzed daily in these individual jars as described previously (Crisosto et al., 1993). A fruit sample of each treatment and cultivar was assessed for flesh firmness, soluble solids concentration (SSC), titratable acidity (TA) and skin and flesh color during ripening at 20°C after harvest at 0 d and every 2 days up to 14 d as described previously (Minas et al., 2013). Fruit skin and flesh color changes were expressed as hue angle (*h*°), ranging from green (0°) through yellow (90°), red (180°), and blue (270°), ending back in dark blue-green (360°) (Crisosto et al., 1997). The experimental setup is presented in Supplementary Figure 1. Statistical analysis was performed as described in Experiment 1 (Section Experiment 1: Softening Segregation).

### Molecular characterization

Genomic DNA was isolated from leaves using the DNeasy Plant Mini Kit (Qiagen, Dusseldorf, Germany) following the manufacturer's instructions. The DNA was quantified and diluted to approximately 25 ng μL^−1^ in water to carry out PCR amplifications. Ten microsatellite markers previously developed in *Prunus* (Table 3) were analyzed for transferability and polymorphism in the plum cultivars tested. These markers were selected for their polymorphism in *Prunus*. PCR reactions were carried out in a total volume of 10 μL containing 5 ng μL^−1^ DNA, 1× Gold Buffer (Applied Biosystems, Inc., Foster City, CA, USA), 2 mM MgCl_2_, 0.2 mM of each dNTP, 0.2 pmol μL^−1^ of each primer and 0.025 units μL^−1^ AmpliTaq Gold DNA polymerase (ABI). PCR cycling conditions for all primers were an initial step of 5 min at 94°C, followed by 30 cycles of 30 s at 94°C, 1 min at 54°C, and 1 min at 72°C, and concluding with 1 cycle of 7 min at 72°C. The DNA amplification products were separated by electrophoresis in 2% agarose gels. Forward microsatellite primers were labeled with three fluorescence dyes including NED, VIC, and 6-FAM, and the size standard was ROX 400HD (Applied Biosystems) for the ABI PRISM 3100. PCR products were run in multiplexes using capillary electrophoresis on an ABI PRISM 3100 Genetic Analyzer. The microsatellite fragment sizes were analyzed using GeneMapper software v4.1 (Applied Biosystems).

**Table 3:** **List of 10 microsatellite markers used to distinguish plum cultivars**.

**SSR^a^**	**Origin**	**LG^b^**	**Size range (bp)**	***A*^c^**	***N*^d^_e_**	***H*^e^_o_**	***H*^f^_e_**	**PIC^g^**	***F*^h^_is_**	**References**
BPPCT001	Peach	2	120–170	17	4.45	0.78	0.84	0.84	0.08	Dirlewanger et al., 2002
BPPCT004	Peach	2	170–210	15	4.50	0.78	0.67	0.67	0.10	Dirlewanger et al., 2002
BPPCT025	Peach	6	120–212	22	5.44	0.82	0.91	0.91	−0.01	Dirlewanger et al., 2002
BPPCT040	Peach	4	120–154	14	6.25	0.84	0.85	0.85	−0.05	Dirlewanger et al., 2002
CPPCT006	Peach	8	170–216	17	5.56	0.82	0.77	0.77	−0.16	Aranzana et al., 2002
CPSCT012	Japanese plum	6	138–180	20	9.10	0.89	0.88	0. 88	0.01	Mnejja et al., 2004
CPSCT026	Japanese plum	7	163–209	21	7.69	0.87	0.90	0. 90	0.01	Mnejja et al., 2004
CPSCT042	Japanese plum	7	159–183	12	6.25	0.84	0.85	0. 85	0.03	Mnejja et al., 2004
PaCITA4	Apricot	3	113–151	11	8.33	0.88	0.77	0.77	−0.14	Dondini et al., 2006
UDP98-412	Peach	6	90–134	21	25	0.96	0.91	0.91	−0.06	Testolin et al., 2000
Average				17.0	8.26	0.84	0.83	0.80	−0.02	

*Crop of origin and references for the ten markers and genetic diversity parameters based on 43 plum cultivars*.

a *Single sequence repeat or microsatellite marker*.

b *Linkage group based on an almond x peach linkage map (Dirlewanger et al., 2004)*.

c *Observed alleles per locus*.

d *Effective number of alleles*.

e *Observed heterozygosity*.

f *Expected heterozygosity*.

g *Polymorphic index content*.

h *Wright's fixation index*.

### Genetic analysis

Ten microsatellite markers were used to analyze the 43 plum cultivars. The number of observed alleles per locus (*A*), effective number of alleles (*N_e_*) (Kimura and Crow, 1964), observed heterozygosity (*H*_o_ = number of heterozygous individuals/number of individuals scored), expected heterozygosity and Wright's fixation index (*F*_is_ = 1 − *H*_o_/*N*_e_) were calculated using PopGene 1.31 software (Yeh et al., 1997). The numerical, two-column marker data were converted to a 0/1 matrix (presence/absence of alleles) to facilitate analysis of polyploidy. Simple likelihood of a random matching profile was calculated by multiplying allele frequencies across loci. Genetic similarity between cultivars was estimated using the coefficient of similarity index (Nei and Li, 1979) calculated using NTSYSpc-2.11 version 2.1 (Rohlf, 2000). A dendrogram was generated using the unweight pair-group method (UPGMA).

### Fruit growth, development and ripening “On-Tree” of the genetically related plums

Fruit growth, development and ripening/softening patterns of the climacteric cultivar “Santa Rosa” and the genetically related, suppressed- and non-climacteric cultivars “Late Santa Rosa,” “Casselman,” “Roysum,” and “Sweet Miriam” started immediately after natural fruit drop, approximately 90 d after full bloom (DAFB), in three randomly selected trees per cultivar. We observed 1 or 2 weeks of difference in the date of full bloom among these 5 cultivars. Fruit growth patterns were monitored weekly in 20 labeled fruits per tree by measuring fruit diameter (size) to estimate fruit volume and flesh firmness as described previously (Grossman and DeJong, 1995). Statistical analysis was performed as described in Experiment 1 (Section Experiment 1: Softening Segregation). Degree-days were calculated using the single sine, horizontal cut-off method with critical temperatures 7 and 35°C (Grossman and DeJong, 1995) through the degree-day calculator of the University of California IPM online platform (http://www.ipm.ucdavis.edu/WEATHER/index.html#DEGREEDAYS).

## Results

### Fruit quality traits

Flesh firmness, SSC, and TA ranged within commercial standards among “Santa Rosa,” “Eldorado,” “Ambra,” “Friar,” “Durado,” “July Santa Rosa,” “Laroda,” “Joanna Red,” “Angeleno,” “Casselman,” “Roysum,” “Late Santa Rosa,” and “Sweet Miriam” plum fruits. These cultivars have been historically and some are still commercially marketed at different dates during the California plum-growing season. During the two seasons of evaluation, plums were harvested at 27–35 N flesh firmness, SSC was 11.1–19.7% and TA varied from 0.30 to 1.60%. The highest SSCs at harvest were in “Joanna Red” (16%), “Casselman” (15.7%), “Angeleno” (16.8%), “Roysum” (15.0%) and “Sweet Miriam” (19.6%) (Table 1).

### Softening segregation

**Figure 1:**
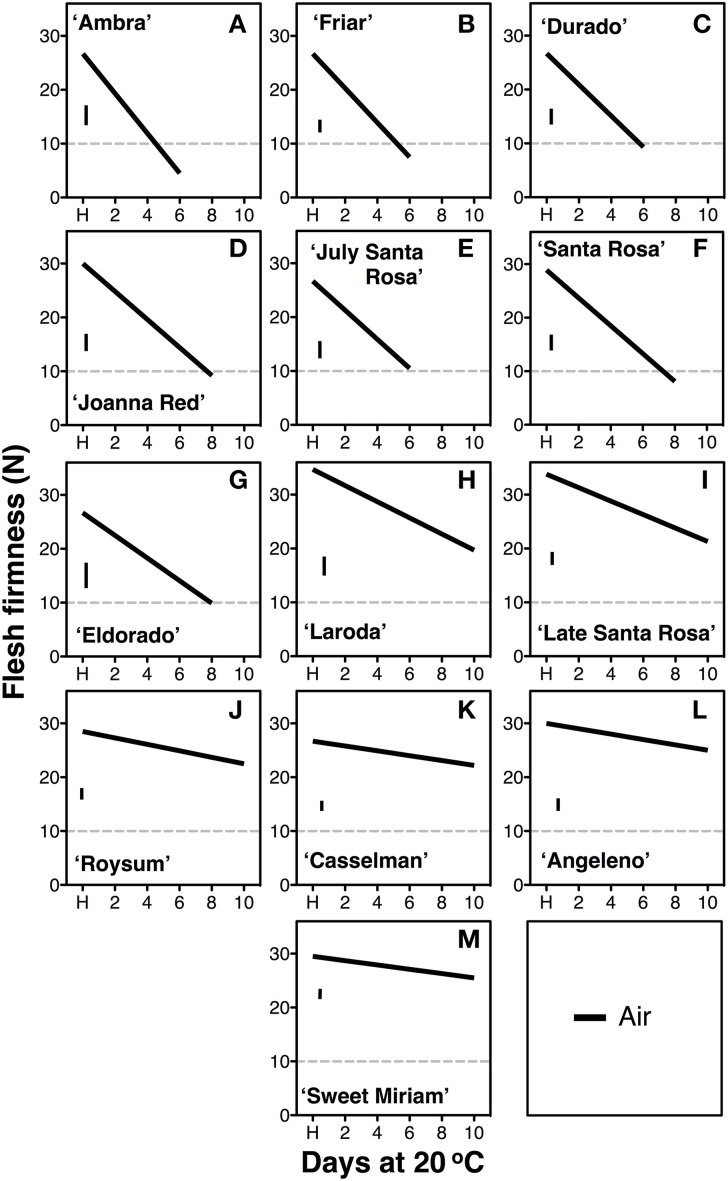
**Softening segregation following harvest**. Softening patterns of thirteen Californian plum cultivars during postharvest ripening at 20°C and 90% RH for up to 10 d immediately after harvest (H). The cultivars segregated into fast-softening (climacteric, **A–G**) and slow-softening (suppressed climacteric, **H–M**) groups according to the softening rate, estimated as flesh firmness loss per day. The horizontal dotted line marks the 10-N flesh firmness threshold of the “ready-to-eat” stage. The vertical bars in each particular figure represent the least significant difference (LSD, *P* = 0.05).

The rate of softening after harvest, measured during ripening at 20°C, varied significantly among cultivars (Figure 1). Seven of the tested cultivars attained the “ready to eat” stage (flesh firmness ≤ 10.0 N) within the 10-d period studied. The fast softening rate of 2.1–3.7 N firmness loss per day (Table 1), measured in “Ambra,” “Friar,” “Durado,” “Joanna Red,” “July Santa Rosa,” “Santa Rosa,” and “Eldorado,” is similar to that observed in most commercial climacteric Japanese plum cultivars (Martinez-Romero et al., 2003; Crisosto and Day, 2011). However, a slow rate of softening was recorded for “Angeleno,” “Casselman,” “Roysum,” “Late Santa Rosa,” “Laroda” and “Sweet Miriam.” In this group, the “ready-to-eat” stage was not reached within 10 d. Among these slow-softening cultivars, “Late Santa Rosa,” and “Laroda” softened faster (1.0–1.8 N firmness loss per d) than the rest of this group, which lost 0.2–0.7 N firmness per day (Table 1).

### Effect of exogenous ethylene on softening rate

**Figure 2:**
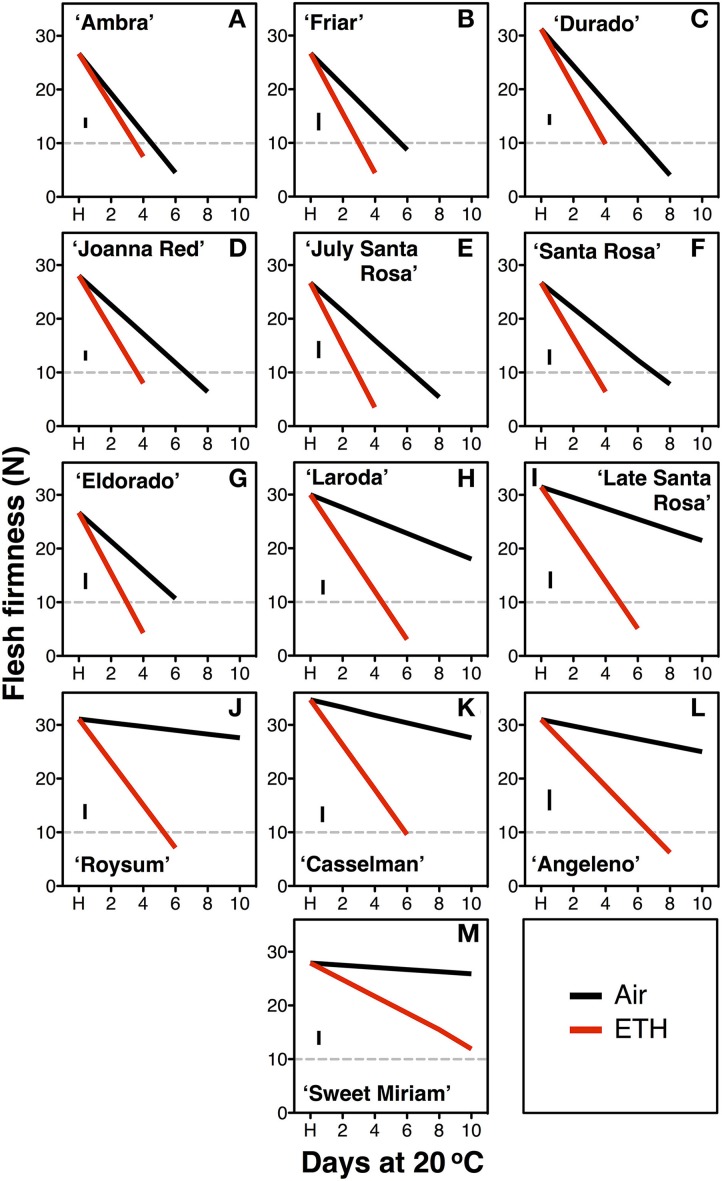
**Segregation of the exogenous ethylene softening response**. Postharvest flesh firmness changes of the fast-softening (climacteric, **A–G**) and slow-softening (suppressed climacteric, **H–M**) plum cultivars during 10 d ripening at 20°C and 90% RH immediately after harvest (H) on fruit exposed or not to exogenous ethylene (ETH, 10 μL L^−1^). The horizontal dotted line marks the 10-N flesh firmness threshold of the “ready-to-eat” stage. The vertical bars in each particular figure represent the least significant difference (LSD, *P* = 0.05).

**Figure 3:**
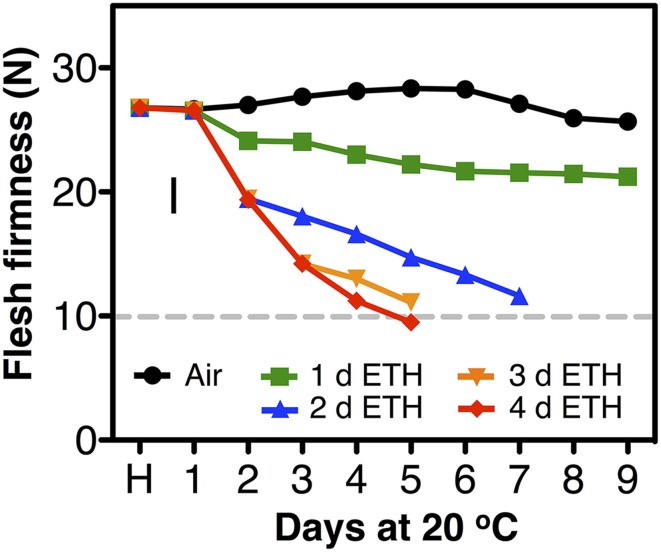
**Postharvest softening response of “Roysum” plums to exogenous ethylene**. Immediate flesh firmness changes following harvest (H) of the suppressed-climacteric “Roysum” plum cultivar in response to exogenous ethylene (ETH, 10 μL L^−1^) for 0, 1, 2, 3, or 4 d and subsequent transfer to ambient (ethylene-free) air for up to 9 d at 20°C and 90% RH. The horizontal dotted line marks the 10-N flesh firmness threshold of the “ready-to-eat” stage. The vertical bar represents the least significant difference (LSD, *P* = 0.05).

In the second season, constant application of exogenous ethylene during ripening increased the softening rate of both fast- and slow-softening cultivars (Figure 2). In fast-softening cultivars, the rate of softening without ethylene was similar to the previous season; these plums reached the “ready-to-eat” stage within 6–8 d. However, in the presence of exogenous ethylene, all cultivars reached the “ready-to-eat” stage approximately 2 d earlier than fruit ripened without ethylene exposure and cold storage. As in the first season, fruit from the six slow-softening cultivars did not reach the “ready-to-eat” stage within 10 d under air. However, when exposed to constant ethylene, all six cultivars reached the “ready-to-eat” stage at different times within the 10-d experiment: 4 d for “Laroda” and “Late Santa Rosa,” 6 d for “Casselman” and “Roysum,” 8 d for “Angeleno,” and 12 d for “Sweet Miriam” (Figure 2). As in the previous season, “Late Santa Rosa” and “Laroda” softened faster than the rest of the slow-softening cultivars, suggesting that further physiological differences in softening regulation exist among the slow-softening cultivars. The need for continuous ethylene during ripening observed in the slow-softening group was demonstrated using “Roysum” plums, in which softening occurred only when ethylene was present. To reach the “ready-to-eat” stage, a 3 to 4 d ethylene treatment was necessary, and upon removal from the ethylene-enriched atmosphere after 1 or 2 d, fruit softening rate slowed significantly or stopped (Figure 3).

During ripening at 20°C, respiration measured as carbon dioxide production rate was 15 ± 5 mg CO_2_ kg h^−1^ for the slow-softening cultivars “Angeleno,” “Late Santa Rosa,” “Casselman,” “Roysum” and “Sweet Miriam.” These respiration rates are almost half of those measured in typical climacteric plums such as “Santa Rosa,” “Friar,” “Ambra,” “July Santa Rosa,” and “Joanna Red” during climacteric peaks. In addition, these slow-softening plums produced very low and/or undetectable endogenous ethylene concentrations without ethylene application and little or no endogenous ethylene after short exposures to exogenous ethylene (data not shown).

### Physiological characterization of three distinct ripening patterns in plum fruit

**Figure 4:**
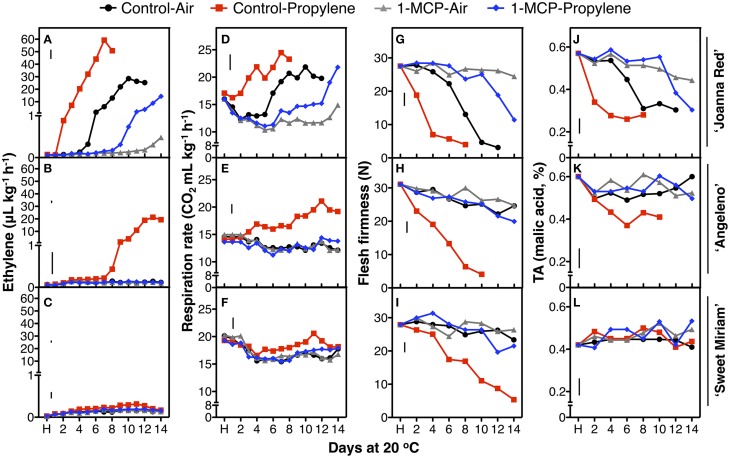
**Postharvest changes during ripening**. Changes in ethylene production **(A–C)**, respiration rate **(D–F)**, flesh firmness **(G–I)** and titratable acidity (TA, **J–L**) of “Joanna Red” **(A,D,G,J)**, “Angeleno” **(B,E,H,K)** and “Sweet Miriam” **(C,F,I,L)** plums, previously treated or not with 1-MCP (0.5 μL L^−1^, 24 h, 20°C), during ripening at 20°C and 90% RH immediately after harvest (H) under air or propylene (500 μL L^−1^). The vertical bars in each particular figure represent the least significant difference (LSD, *P* = 0.05).

**Figure 5:**
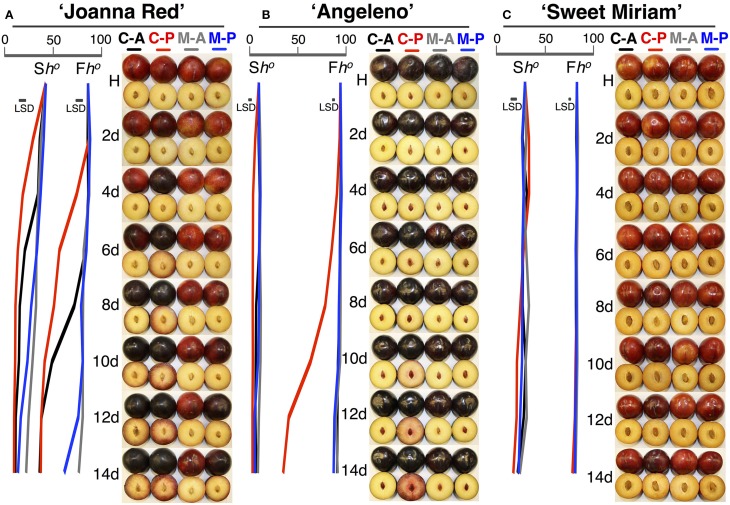
**Color changes during ripening following harvest**. Skin and flesh color changes of “Joanna Red” **(A)**, “Angeleno” **(B)**, and “Sweet Miriam” **(C)** plums, previously treated or not with 1-MCP (0.5 μL L^−1^, 24 h, 20°C), during ripening at 20°C and 90% RH immediately after harvest under air or propylene (500 μL L^−1^). The horizontal bars in each particular figure represent the least significant difference (LSD, *P* = 0.05). Abbreviations: S*h°*, skin color (Hue angle, *h°*); F*h°*, flesh color (Hue angle, *h°*); H, harvest; C-A, control-air; C-P, control-propylene; M-A, 1-MCP-air; M-P, 1-MCP-propylene.

To characterize in detail the distinct softening patterns observed in the first two seasons, a fast-softening (“Joanna Red”) and two slow-softening cultivars (“Angeleno” and “Sweet Miriam”) were selected and treated with propylene and 1-MCP to dissect the different ripening types in plum fruit. Untreated, air-ripened (C-A) “Joanna Red” plums showed typical climacteric ripening. “Joanna Red” C-A-treated plums showed a climacteric ethylene production peak after 10 d ripening at 20°C, and propylene (C-P) induced a two-fold greater peak that occurred 3 d earlier than the C-A (Figure 4). 1-MCP-treated and air-ripened (M-A) fruit had no climacteric ethylene production while 1-MCP-treated and propylene-ripened (M-P) fruit produced the most ethylene after 14 d (50% less than C-A). Respiration rate peaked in C-A plums after 10 d ripening at 20°C, while in C-P peaked 3 d earlier. 1-MCP treatment inhibited the respiration rate of air-ripened plums (M-A) by 40–50% and no climacteric peak was observed. On the other hand, M-P plums had a respiration rate peak at 14 d, which was equal to that of C-A fruit. During ripening, C-P fruit softened rapidly to the “ready-to-eat” stage after 4 d, while C-A plums reached this point after 9 d. 1-MCP treatment dramatically inhibited softening of air-ripened fruit, but protected propylene-ripened plums from rapid softening. Titratable acidity (TA) was reduced during ripening in all treatments; however, this phenomenon was advanced by propylene and reduced by 1-MCP (Figure 4). The skin and flesh colors of untreated plums changed during ripening from red to dark red and from yellow to red, respectively, while the observed color changes were accelerated by propylene and delayed by 1-MCP (Figure 5). SSC remained generally stable in all treatments (Supplementary Figure 2), but propylene increased the ratio of SSC to TA during ripening by reducing TA (Figure 4, Supplementary Figure 3).

On the other hand, “Angeleno” plums exhibited suppressed-climacteric ripening behavior. The climacteric ethylene peak in untreated propylene-ripened (C-P) plums occurred after 13 d ripening and was about half that of “Joanna Red” plums (Figure 4). The other treatments did not affect ethylene production during ripening after harvest. The respiration climacteric peak in C-P plums was detected at 12 d, while no peak was observed in the other treatments. The postharvest softening of C-P “Angeleno” plums occurred at a significantly slower rate than “Joanna Red” plums. C-P fruit started softening at 2 d and reached the “ready-to-eat” stage at 8 d, while plums from other treatments remained firm (>20 N) and never reached this degree of softening during ripening. TA decreased during ripening after harvest in C-P fruit only, while these parameters remained unaffected in the rest treatments (Figure 4). Propylene induced skin color darkening in untreated and 1-MCP-treated fruit at 2 and 12 d, respectively, while it induced flesh color reddening at 4 and 14 d, respectively. No changes were observed in air-ripened plums (Figure 5). SSC remained stable in all treatments (Supplementary Figure 2), while the SSC:TA ratio increased in C-P fruit only; however, this SSC:TA increase was significantly smaller than of “Joanna Red” plums ripened under the same conditions (Supplementary Figure 3).

In contrast, “Sweet Miriam” plums exhibited non-climacteric ripening behavior: ethylene production remained at basal levels under both air and propylene and with and without 1-MCP during ripening after harvest (Figure 4). No respiration climacteric was observed in “Sweet Miriam” plums: there were no differences in CO_2_ production rates among treatments during ripening, except in C-P treated fruit, where there was a weak increase in CO_2_ production rate at 11 d. There were postharvest changes in flesh firmness and skin color of “Sweet Miriam” only in C-P treated fruit; however, these changes occurred at a significantly slower rate than in “Joanna Red” or “Angeleno” plums. In C-P plums, fruit softening began after 6 d and reached the “ready-to-eat” stage after 12 d ripening, while plums from the other treatments remained firm (flesh firmness > 20 N, Figure 4). TA was unaffected by postharvest ripening in all treatments (Figure 4). Air-ripened plums exhibited no skin color change, while propylene slowly advanced the skin color change of C-P fruit from red to slightly darker red during ripening (Figure 5). Flesh color of the C-P fruit did not change during ripening (Figure 5) and SSC remained stable during ripening in all treatments (Supplementary Figure 2). Because “Sweet Miriam” plums had both high SSC and low TA, they showed the highest SSC:TA ratio at harvest among the cultivars tested (Table 1, Supplementary Table 1). This ratio was not affected by either ripening atmosphere or treatment and remained stable during ripening (Supplementary Figure 3).

### Genetic diversity assessed by microsatellite polymorphism

Amplification of the 10 microsatellite markers was successful for all 43 plum cultivars analyzed in the present study (Table 3). Genotypes showing a single band were considered homozygous for that particular locus. The cumulative marker profiles were unique to each of the 43 cultivars with the exception of one group of six apparently identical cultivars. A total of 170 alleles were observed. The mean number of alleles per marker was 17; BPPCT025 detected the most alleles (22) and PaCITA4, the fewest (11) (Table 3). Observed heterozygosity (*H*_o_) ranged from 0.78 (BPPCT001 and BPPCT004) to 0.96 (UDP98-412), with an average of 0.84 across all 10 markers (Table 3). Expected heterozygosity (*H*_e_) ranged from 0.67 (BPPCT004) to 0.91 (BPPCT025 and UDP98-412) and averaged 0.83 across all 10 markers. Comparing *H*_e_ to *H*_o_, the fixation index (*F*) was near zero across all 10 markers. The marker BPPCT004 showed a marginal excess of heterozygotes and BPPCT025 minimal dearth (*F* = −0.16 and 0.10, respectively). The high values obtained for the number of alleles, *H*_o_ and *H*_e_ indicate wide genetic diversity within this group of plum cultivars and demonstrate the efficacy of this set of 10 markers in uniquely identifying sexually-derived plum cultivars. The six most informative markers, based on *H*_e_, are more than sufficient to distinguish each of the 38 unique genotypes within this study set. With these six markers alone, based on allele frequencies from this study set, the likelihood of obtaining a new individual that possesses the single profile shared by “Santa Rosa,” “July Santa Rosa,” “Late Santa Rosa,” “Casselman,” “Roysum,” and “Sweet Miriam” through a sexual cross is less than one in one hundred billion. A shared profile is, however, very consistent with five of these cultivars being somatic mutations or “bud sports” that trace back to one parent variety, which historic records identify as “Santa Rosa.”

### Genetic similarity

**Figure 6:**
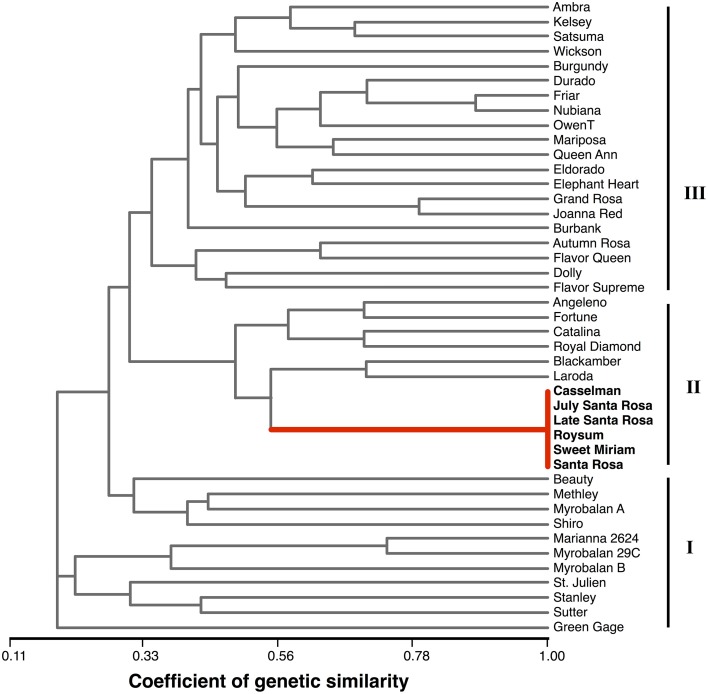
**Phylogenetic dendrogram**. UPGMA tree showing the genetic relationships among the 43 plum cultivars analyzed in the study. Microsatellite marker scores presented in Supplementary Table 2.

Based on the ten polymorphic markers, a similarity matrix was calculated and the relationships among the 43 plum cultivars were presented as a UPGMA dendrogram (Figure 6). The genotypes grouped into three main clusters. The first cluster, “Green Gage” to “Beauty,” is a loosely associated group of hybrids derived primarily from species other than *P. salicina* and is divided into two sub-clusters. The initial sub-group contains three *P. domestica* cultivars, “Green Gage,” “Stanley” and “Sutter,” a dried plum/prune cultivar, and the rootstock cultivars “Marianna” and “Myrobalan,” which are derived from *P. cerasifera* and/or *P. munsoniana*. The second sub-group contains the complex hybrids “Beauty,” “Shiro,” and “Methley.” “Myrobalan A” is a misnamed selection of unknown origin. The middle cluster is a well-defined, closely related group of cultivars. It contains “Santa Rosa,” the five “Santa Rosa”-derived somatic mutants (“July Santa Rosa,” “Late Santa Rosa,” “Casselman,” “Roysum,” and “Sweet Miriam”) and six other cultivars breed by Luther Burbank with “Santa Rosa” and “Queen Ann” as common ancestors (Okie, 1995; Okie and Ramming, 1999). The third cluster primarily contains the non-“Santa Rosa”-derived branch of Burbank's plum releases and is consistent with breeding records. For example, “Queen Ann” is grouped with its parent “Eldorado,” sibling “Nubiana” and half sib “Grand Rosa”; “Friar” is a progeny of “Nubiana” and parent of “Blackamber.” Thus, the microsatellite system used here is sufficient to distinguish among even closely related genotypes, but does not discriminate bud sport mutations within a cultivar.

### Fruit growth, development, and ripening “On-Tree”

**Figure 7:**
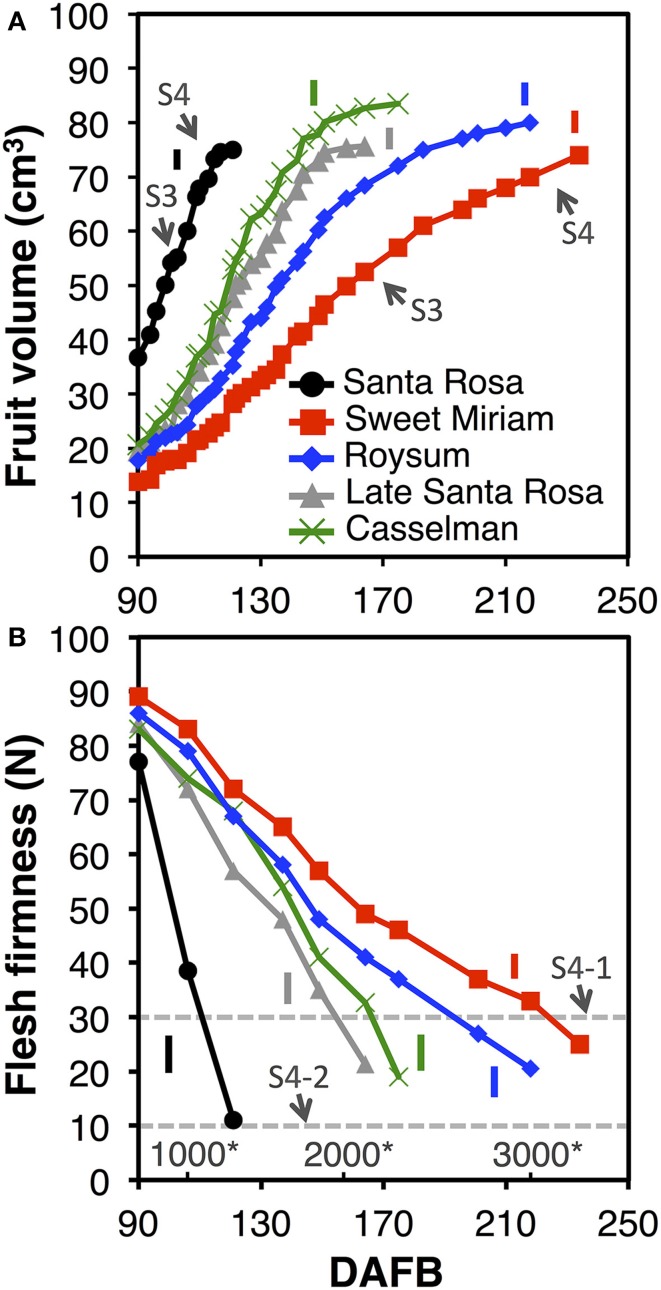
**Plum fruit development, maturation and ripening “on-tree.” Fruit volume (A)** and flesh firmness **(B)** changes during maturation and ripening “on-tree” of the California plum cultivar “Santa Rosa” and the “Santa Rosa”-derived bud sport mutants: “Late Santa Rosa,” “Roysum,” “Casselman,” and “Sweet Miriam,” Arrows indicate the developmental stages defined in the text. The horizontal dotted line marks the 30- and 10-N thresholds of the commercial maturity (S4-1) and “ready-to-eat” (S4-2) developmental stages (Crisosto, 1994). Numbers with asterisks at the bottom of the plate **(B)** indicate the degree-days after full bloom (Grossman and DeJong, 1995). The vertical bars in each particular figure represent the least significant difference (LSD, *P* = 0.05).

Fruit growth, development and ripening “on-tree” were monitored in the climacteric cultivar “Santa Rosa,” its suppressed-climacteric bud-sports “Late Santa Rosa,” “Casselman” and “Roysum,” and its non-climacteric bud-sport “Sweet Miriam.” Plum fruit growth can be divided into four stages: pit-hardening (Stage 2, S2), second exponential growth phase (Stage 3, S3), full red color (commercial harvest, S4-1) and fully ripe (“ready to eat,” S4-2). The lengths of the growth stages were determined based on fruit volume and expressed as a function of days after full bloom (DAFB) and degree-days after full bloom (Figure 7A) (Chalmers and Ende, 1975; Grossman and DeJong, 1995; Tonutti et al., 1997). In this study, data collection started at the last phase of pit-hardening (S2) to avoid natural fruit drop within the labeled fruit. Although final fruit size was similar in all cultivars at commercial harvest, “Santa Rosa” fruits reached S4-2 at 121 DAFB as determined by flesh firmness (Crisosto, 1994) (Figure 7B), “Late Santa Rosa” fruits needed 164 DAFB, “Casselman” fruits needed 175 DAFB, “Roysum” fruit needed 218 DAFB, and “Sweet Miriam” fruits needed 234 DAFB. All suppressed-climacteric and non-climacteric cultivars had longer S3 and S4 periods than the climacteric “Santa Rosa.” For example, while the second exponential growth phase (S3) lasted 80–85 DAFB in “Sweet Miriam,” it only required 19–21 DAFB in “Santa Rosa” fruits (Figure 7A). Harvest date in this group is mainly controlled by fruit ripening rather than the length of the pit-hardening stage (S2), as most bloomed within 7–14 d of each other (Figure 7; Supplementary Table 3). In all cultivars, flesh firmness declined during fruit maturation “on-tree”; however, fruit hanging “on-tree” had a different softening rate (Figure 7B). During the last phase of fruit development (S4), “Santa Rosa” fruits reached the full red color/commercial harvest stage (flesh firmness ~30 N, S4-1) in 110 DAFB (1086 degree-days after full bloom) and after 12 d softened rapidly to the “ready-to-eat” stage (flesh firmness ~10 N, S4-2). In contrast, the suppressed- and non-climacteric fruit softened very slowly “on-tree,” taking 151, 157, 193, and 212 DAFB or 1850, 1958, 2631, and 2929 degree-days for “Late Santa Rosa,” “Casselman,” “Roysum,” and “Sweet Miriam” fruits, respectively, to reach the commercial harvest (flesh firmness ~30 N, S4-1), while they never reached the S4-2 stage (~10 N) during our evaluation period (Figure 7B).

## Discussion

### Physiological characterization of three distinct ripening patterns in plum fruit

Historically, Japanese plums have been classified as a climacteric fruit; however, some cultivars behave as suppressed-climacteric: characterized by extremely slow softening accompanied by low respiration rate and endogenous ethylene production during postharvest ripening (Abdi et al., 1997; Martinez-Romero et al., 2003; Candan et al., 2008). These distinct ripening behaviors were confirmed in our present study among 13 commercial Californian plum cultivars that segregated into two main groups based on their softening rates: normal- or fast-softening (climacteric) and slow-softening (suppressed- and non-climacteric). The cultivars were further characterized based on their softening responses under continuous exogenous ethylene. Ethylene exposure uncovered significant differences among the slow-softening cultivars, with some responding faster than others and some having an extremely slow softening response, suggesting that exogenous propylene or ethylene exposure is a useful tool to distinguish the slow-softening ripening trait. Ethylene or propylene rapidly accelerates ripening in climacteric fruit in a very constant manner (McMurchie et al., 1972; Lelievre et al., 1997), while 1-MCP inhibits ripening in most of this type of fruit (Watkins, 2006). A non-climacteric fruit's response to exogenous ethylene or propylene is slow or non-existent, and ripening is considered an ethylene-independent process (McMurchie et al., 1972; Lelievre et al., 1997). However, several exceptions to this rule have been reported (Lelievre et al., 1997). To distinguish the different ripening patterns observed among the plum cultivars tested, a fast-softening cultivar (“Joanna Red”) and two slow-softening cultivars (“Angeleno” and “Sweet Miriam”) with similar harvest dates were selected and treated with propylene and 1-MCP. Propylene simulated the effect of exogenous ethylene on fruit ripening but allowed the fruit's endogenous ethylene production to be monitored during treatment; the concentration used (500 μL L^−1^) was previously shown to accelerate plum ripening (Abdi et al., 1997).

The detailed physiological characterization revealed three distinct ripening patterns in plum fruit for first time: climacteric, suppressed-climacteric and non-climacteric. “Joanna Red” plums behaved as typical climacteric fruits during ripening as previously reported (Manganaris et al., 2008), as did “Santa Rosa” (Martinez-Romero et al., 2003), “Ambra,” “Friar,” “July Santa Rosa,” “Durado” and “Eldorado,” based on softening patterns during ripening in air or in response to exogenous ethylene (Figures 1, 2). Their ripening behavior was characterized by a tremendous climacteric spike in ethylene production, rapid softening, loss of TA, and changed skin and flesh color from light red and light yellow, respectively, to dark red. Propylene strongly accelerated these physiological changes, while 1-MCP inhibited them (Figures 4, 5).

“Angeleno” plums behaved as a suppressed-climacteric fruit as previously reported for this cultivar (Candan et al., 2008; Singh and Khan, 2010) or others like “Shiro” (Abdi et al., 1997; El-Sharkawy et al., 2007), “Rubyred” (Abdi et al., 1997), “Golden Japan” (Martinez-Romero et al., 2003), and “Amber Jewel” (Singh and Khan, 2010). This type of plum showed no important postharvest changes during 14 d after harvest at 20°C, except for fruit exposed to propylene (C-P), which exhibited a climacteric increase of ethylene production, softening, loss of TA and changes in skin and flesh color from dark purple and light yellow to dark black and red, respectively (Figures 4, 5). Untreated, air-ripened fruit softened and the skin and flesh color changed at 25 after harvest at 20°C (data not shown). The postharvest changes during ripening under propylene in this cultivar occurred more slowly than in the climacteric cultivars. Ethylene production peaks were half that of “Joanna Red” plums, the climacteric increase in ripening was less sharp, and the softening rate was slower. 1-MCP inhibited postharvest changes in “Angeleno” plums, but its impact on propylene-treated fruit was more obvious due to the slow rate of the changes under air. Plum cultivars which exhibit slow softening, like “Laroda,” “Late Santa Rosa,” and “Roysum” should also be classified as suppressed-climacteric based on their softening rates during ripening in air or in response to exogenous ethylene treatment (Figures 1, 2). “Casselman” plums have also been previously reported to produce low concentrations of endogenous ethylene in response to exogenous ethylene (Crisosto et al., 1993). It is noteworthy that according to our “intermittent test,” this group of plums requires a minimum 3 d (72 h) ethylene exposure during postharvest ripening to soften to an “eating-ripe” firmness, since softening slowed upon removal of the fruit to an ethylene-free atmosphere after only 24 or 48 h exogenous ethylene exposure.

The ripening of “Sweet Miriam” plums was characterized by tremendously slow postharvest physiological changes, even under propylene. The only postharvest change occurring in this cultivar was a very slow rate of softening and skin color change in untreated fruit under a continuous supply of propylene, but there was no endogenous ethylene production (Figures 4, 5). “Sweet Miriam” plums did not soften during ripening “off-tree” without exogenous ethylene or propylene. No TA changes were found even under propylene in “Sweet Miriam” plums, unlike in the climacteric and suppressed-climacteric plums, in which the organic acids may provide substrates for the increased respiration.

Different responses to exogenous ethylene have been reported for several non-climacteric fruits. In citrus fruits and pineapples, degradation of chlorophyll was reported, while treatment with 1-MCP inhibited ethylene responses and delayed senescence symptoms (Porat et al., 1999; Selvarajah et al., 2001). In strawberry, postharvest exogenous ethylene induced climacteric-like responses such as increased softening rate, accumulation of red pigments and up-regulation of ethylene receptor genes (Tian et al., 2000; Trainotti et al., 2005). Similarly, in grape berries, synthesis of anthocyanins and aromatic volatiles and loss of TA increased in response to exogenous ethylene, while 1-MCP inhibited ethylene action and subsequent ripening changes (Chervin et al., 2004). Similarly, slow responses to external ethylene have been reported in non-climacteric pleiotropic tomato mutants such as rin, which responds to treatment with exogenous ethylene or propylene with increased ripening rate, slow softening, and yellow color development while ethylene production remains at basal levels (Herner and Sink, 1973; McGlasson et al., 1975). Additionally, different stony hard peach cultivars produce low endogenous ethylene concentrations during ripening and soften slowly, but both endogenous ethylene biosynthesis and softening are stimulated upon exposure to propylene or ethylene (Tatsuki et al., 2006; Begheldo et al., 2008).

The critical difference between climacteric and non-climacteric fruits rests in their relative abilities to perceive and produce ethylene in response to exogenous ethylene or propylene (McGlasson et al., 1975). On the basis of similar responses of “Sweet Miriam” plum, citrus fruit, strawberry and rin tomato to exogenous propylene, we conclude that the fruits of this cultivar are non-climacteric; such behavior is confirmed here for the first time in plum fruit. The possible differences in mRNA accumulation patterns associated with ethylene perception and signal transduction components, such as *ETR1, ERS1* and *CTR1*, the ACC-synthase gene family (*ACS1, ACS3a, ACS3b, ACS4*), and the ethylene-responsive transcriptional factors (*ERFs*) among non-climacteric, suppressed-climacteric and typical climacteric plum cultivars should be tested using this group of cultivars with similar harvest dates and genetic background.

### Non-climacteric “Sweet Miriam” is a bud sport mutation of the climacteric plum cultivar “Santa Rosa”

Following characterization of the novel, non-climacteric ripening behavior of “Sweet Miriam” plums, it became important to demonstrate that “Sweet Miriam” originated as a somatic mutation of the climacteric cultivar “Santa Rosa,” as reported for several other slow-softening plum cultivars such as “Late Santa Rosa,” “Casselman,” and “Roysum” (Okie, 1995; Okie and Ramming, 1999). Somatic mutants, or bud-sports, are generated by somatic cell mutations in the meristematic layers from which a new shoot is derived. Infrequently, such mutations result in phenotypic changes in the shoots of woody perennial plants (Marcotrigiano, 1997; Walker et al., 2006). Bud-sports with desirable phenotypes are maintained and marketed through vegetative propagation and serve as an important source of variability. The small mutations that lead to bud-sports are only rarely observable within the non-coding DNA associated with microsatellite markers (Riaz et al., 2002). Multi-locus SSR profiles of bud-sports, therefore, are nearly always identical to that of the original seedling.

To reveal any genetic relationships among plum cultivars with distinct ripening behavior, a set of 43 plum cultivars were genetically characterized using SSR analysis (Table 2), including the thirteen cultivars physiologically analyzed in this study. The high transferability of microsatellite loci across the genus *Prunus* observed here is consistent with previous works (Cipriani et al., 1999; Dangl et al., 2009). The number of alleles per locus (17.0) and expected heterozygosity (0.85) obtained in the present population (Table 3) are equal to (Carrasco et al., 2012) or greater than previous studies on *Prunus* populations (Casas et al., 1999; Ahmad et al., 2004; Mnejja et al., 2004; Dangl et al., 2009; Font i Forcada et al., 2012). The diversity we found in plum was greater than that of other *Prunus* species: peach (Font i Forcada et al., 2012), almond (Fernández i Martí et al., 2014), apricot (Hormaza, 2002; Ruthner et al., 2006) or sweet cherry (Fernández i Martí et al., 2012). The high heterozygosity in plum may be due to the self-incompatibility of different *Prunus* species. Almond and Japanese plums are outcrossing species due to the existence of a strong gametophytic self-incompatibility system, and therefore maintain high variability, while peach is less variable because of selfing, a consequence of its self-compatibility (Miller et al., 1989).

Genetic distance analysis grouped the 43 plum samples into three main clusters with varying amounts of sub-clustering (Figure 6). The main clusters were completely consistent with phylogenetic expectations and breeding records. The first cluster contained cultivars derived from species other than *P. salisina*. This group is only loosely associated, reflecting the species-level differences, in contrast to the other two clusters that were both primarily *P. salisina*. The dendrogram also accurately depicts the identity of the “Santa Rosa” bud-sports and the close relationship among cultivars derived from the sexual lineage of “Santa Rosa.”

The results of the genotyping study confirm our hypothesis that the non-climacteric cultivar “Sweet Miriam” is derived from the somatic lineage of the climacteric cultivar “Santa Rosa.” We also confirm a similar somatic lineage reported for several other California plum cultivars, though analysis of microsatellite markers cannot determine the exact order of the somatic lineage. The suppressed-climacteric cultivar “Late Santa Rosa” is presumed to be a bud-sport directly from “Santa Rosa,” while the suppressed-climacteric cultivars “Casselman” and “Roysum” are thought to be derived from “Late Santa Rosa,” as is the case of the climacteric cultivar “July Santa Rosa” (Okie, 1995; Okie and Ramming, 1999).

### Ethylene regulation extends “On Tree” and “Off Tree” ripening increasing fruit quality

Consumption of plums has remained steady or even decreased over the last 15 years, mainly due to lack of flavor at the time of consumption (Crisosto et al., 2004). Consumer acceptance of high-acid plums was significantly lower than that of low-acid plums, due to the low SSC:TA ratio in such fruit (Crisosto et al., 2004; Minas et al., 2013). In contrast to the tomato mutants and transgenic knockdown lines that have been used widely as model systems for ethylene studies, the non-climacteric plum genotype described here is already commercially grown and characterized by a high SSC and SSC:TA ratio (>40; Table 1; Supplementary Table 1), which is linked to high consumer acceptance of plums (Crisosto et al., 2004, 2007; Minas et al., 2013). Our hypothesis is that the consumer quality and flavor of plums can be improved if fruit can remain “on-tree” longer (Figure 7), because of increased accumulation of sugars and nutrients. This longer “on-tree” ripening period can produce a more uniform maturity of the fruit within the canopy, which in turn will allow less frequent picking, save hand labor costs and potentially allow mechanical harvesting. Using non-climacteric plum cultivars, the stone fruit industry may reduce postharvest losses during retail handling and may potentially avoid cold storage and its associated disorders, because these plum types soften more slowly “on-” and “off-tree” than climacteric cultivars.

## Conclusions

Although fruit ripening has generally been studied using tomato and its mutations as a model, the breakthrough discovery of a non-climacteric plum bud-sport described here is of great importance because stone fruits are economically important and they provide new opportunities to dissect specific mechanisms of tree-fruit ripening. The non-climacteric cultivar “Sweet Miriam” and the group of suppressed-ripening plums, all bud-sports tracing back to the climacteric cultivar “Santa Rosa,” could expand future stone-fruit research to high-throughput molecular studies based on comparing a “non-ripening” mutant vs. its wild type. This could allow us to study the biological basis of climacteric and non-climacteric ripening in tree-fruits and the key mechanisms governing fruit ripening and senescence, particularly the role of ethylene. Molecular characterization of the genetic relationships described here will assist stone-fruit breeders to select cultivars with fruit that can remain “on-tree” longer to accumulate more sugars, achieve uniform canopy maturity, reduce picking costs, potentially replace the need for cold storage during postharvest handling, and provide high quality fruit to consumers.

### Conflict of interest statement

The authors declare that the research was conducted in the absence of any commercial or financial relationships that could be construed as a potential conflict of interest.
