# Supplementary material for: Discovery of non-climacteric and suppressed climacteric bud sport mutations originating from a climacteric Japanese plum cultivar (Prunus salicina Lindl.)
Source: Front Plant Sci. 2015 May 12;6:316. doi: 10.3389/fpls.2015.00316 (PMC4428209; doi:10.3389/fpls.2015.00316)
Supplement: Supplementary file 1 [file DataSheet1.PDF]

Supplementary Material

Discovery of non-climacteric and suppressed-climacteric bud sport mutations originating from a climacteric Japanese plum cultivar (*Prunus salicina* Lindl.)

Ioannis S. Minas[1], Carolina Font i Forcada[1], Gerald S. Dangl[2], Thomas M. Gradziel[1], Abhaya M. Dandekar[1] and Carlos H. Crisosto[1]*

[1]Department of Plant Sciences, University of California, Davis, CA, United States
[2]Foundation Plant Services, University of California, Davis, United States

***Correspondence:** Carlos H. Crisosto, Department of Plant Sciences, University of California, Davis, One Shields Avenue, CA 95616, United States
chcrisosto@ucdavis.edu

1. Supplementary Tables and Figures

1.1. Supplementary Tables

Supplementary Table 1 | Plum fruit quality traits. Harvest date, physicochemical and qualitative attributes of plum cultivars used in the present study. Values represent the mean of three replications of five fruits.

Cultivar	Harvest date	Firmness (N)		SSC[b] (%)		TA[c] (malic acid, %)		SSC:TA		Skin color (h^o)		Flesh color (h^o)	
Joanna Red	August 17	27.54	a[a]	16.07	c	0.57	a	28.15	b	42.56	a	86.01	b
Angeleno	September 8	31.10	a	16.90	b	0.59	a	28.82	b	9.53	c	94.21	a
Sweet Miriam	October 12	27.90	a	19.73	a	0.42	b	47.42	a	29.52	b	82.97	b

[a]Mean values in the columns followed by the same letter are not statistically significant according to LSD (*P=0.05*).
[b]Soluble solids concentration (%).
[c]Titratable acidity.

Supplementary Table 2 | SSR markers scores.

Cultivar	SSR									
	BPPCT001	BPPCT025	CPSCT012	UDP98-412	BPPCT004	BPPCT040	CPSCT042	CPSCT026	PaCITA4	CPPCT006
Ambra	120 /122	154/160	164	104/120	184/188	130/136	173/175	177/191	129/133	186/194
Angeleno	122 /132	160/188	152/164	108/132	184/186	130/136	163/173	177/179	129/139	186/194
Autumn Rosa	122 /138	150/170	146/174	126/134	186/188	132	179	175/191	129/139	186/194
Beauty	122 /138	150/184	152/178	122/128	186/190	130/136	175/181	171/177	127/137	196/210
Blackamber	122 /138	160/188	152/164	108/122	184/188	130/136	165/173	177/191	127/139	196
Burbank	122 /166	158/186	148	128/132	186/190	130	173/175	177/191	129	186
Burgundy	132 /138	186	164/178	124/132	188/190	130/138	175/181	177/199	129	186
Casselman	120/122	152/160	152/174	108/128	184	132/136	165/179	179/193	127/139	194
Catalina	122 /138	152/188	164/174	128/132	184/186	130/132	163/179	179/193	129/139	186/194
Dolly	122 /132	150/186	148/152	118/128	186/188	132	163/175	179/193	127/133	186/194
Durado	138 /166	152/188	148/174	120/128	186/188	130/136	173	177/193/195	127/129	186/194
Eldorado	132 /138	172/188	146/164	122/132	186	130/138	173/181	177/199	129	186
Elephant Heart	138 /166	160/188	148/152	94/130	188	130/132	173/179	193/199	127/129	186/194
Flavor Queen	138	150/170	146/174	128/132	188	132/146	163/179	171/179	127/139	186
Flavor Supreme	132 /138	170	148/178	120/124	188	130/132	163/179	179/209	129/133	186/194
Fortune	122 /132	160/190	152/164	108/122	184/186	130/132	179/181	179/199	129/139	186/194
Friar	138	188	164	122/132	186/188	130/136	173	177/191	127/129	186/194
Grand Rosa	122 /138	160/170	146/152	124/128	186/188	136/138	173/179	177/193	129/139	186/194
Green Gage	128/136/144 /168	158/168	152/166	90/98		126134		163/189/	113/129	180/186
Joanna Red	122 /138	160/170	146/164	106/128	186/188	136	173/179	177/193	129	186/210
July Santa Rosa	120/122	152/160	152/174	108/128	184	132/136	165/179	179/193	127/139	194
Kelsey	120 /138	154/188	148/164	124/130	184/188	130/132	173/175	175/191	127/129	186/210
Laroda	120 /122	160/188	152/164	108/122	184	136	163/179	177/179	127/139	196/210
Late Santa Rosa	120 /122	152/160	152/174	108/128	184	132/136	165/179	179/193	127/139	194
Marianna 2624	120 /136/154	120/154/164	142/152/156	94/104	172/184/186	130/142/146	171/175/181	171/177/183	129/133 /147	170/186 /194
Mariposa	138	172	146/178/180	124/132	186/188	130/132	179/181	175/209	127/129	186

Methley	138	160/170	146/152	92/122	188	130/148	167/173	171/191	129/139	174/194
Myrobalan A	136 /138	180/182	146/152	108/122	174/186	122/148	173	171	137/143	190/194
Myrobalan B	144 /152	188	152	96	174/184	122/146	171/173	171	129/137	194/200
Myrobalan 29C	136 /152/154	120/164/188	152/156	94/104/108	170/172/186	142/146/148	171/173/175	171/183	129/133 /137	170/186 /194
Nubiana	138	172/188	146/164	122/132	186/188	130	173/179	177/191	127/129	186/194
Owen T	138	154/184	142/164	104/122	186/188	130/136	173	177/191	129	186/210
Queen Ann	138	154/172	142/146	104/132	186/188	130/136	179/181	177	127/129	186/194
Royal Diamond	122 /138	152/186	164/174	128/132	186/190	130/136	163/181	179/199	129/139	182/194
Roysum	120 /122	152/160	152/174	108/128	184	132/136	165/179	179/193	127/139	194
Santa Rosa	120 /122	152/160	152/174	108/128	184	132/136	165/179	179/193	127/139	194
Satsuma	120 /138	172/188	148/164	122/130	184/186	130	173/175	191	129/133	186/194
Shiro	136 /138	154/160	148/152	94/124	186/188	132/154	173	171/191	127/137	194/196 /210/212
St. Julien	122/136/140 /170	162/170/184 /202	150/152/158 /160/168	92/100/128	176/184/188 /210	124/128/132 /134/146	169/175/177 /181	173/183/185 /201/203 /205	129/133	188/194 /198/200 /214
Stanley	136/140/142 /168	154/158/170 /182/212	138/152/158 /166	92/102/108	184/186	126/132/144 /146	171/173/175	165/181/193 /199	129/147	180/186 /190/194 /198
Sweet Miriam	120 /122	152/160	152/174	108/128	184	132/136	165/179	179/193	127/139	194
Sutter	122/134/138 /142/148/166	158/162/168 /170/180	138/144/154 /166	92	174/182/186 /190/208	120/126/128 /132/144	175/179	165/183/199 /205	129/135 /147	188/192
Wickson	122 /138	154	164/176/178	108/124	184/188	132/136	175/179	175/193	127	186/194

Supplementary Table 3 | Fruit developmental stages of the genetically related Californian plum cultivars during maturation and ripening on the tree.

Developmental Stage	Description	DAFB[a] in Santa Rosa	DAFB in Late Santa Rosa	DAFB in Casselman	DAFB in Roysum	DAFB in Sweet Miriam
S3	2nd exponential growth phase	92 – 108 (16)[b]	109 – 142 (33)	101 – 144 (43)	121 – 175 (54)	121 – 192 (70)
S4-1	Full red color stage	108 – 112 (4)	142 – 160 (18)	144 – 170 (26)	175 – 210 (35)	192 – 230 (38)
S4-2	Fully-ripe stage	112 – 121 (7)	160 – 164+ (4+)	170 – 174+ (4+)	210 – 218+ (8+)	230 – 234+ (4+)

[a]Days after full bloom.
[b]Values in parenthesis indicate the length (days) of the specific developmental stage.

1.2. Supplementary Figures

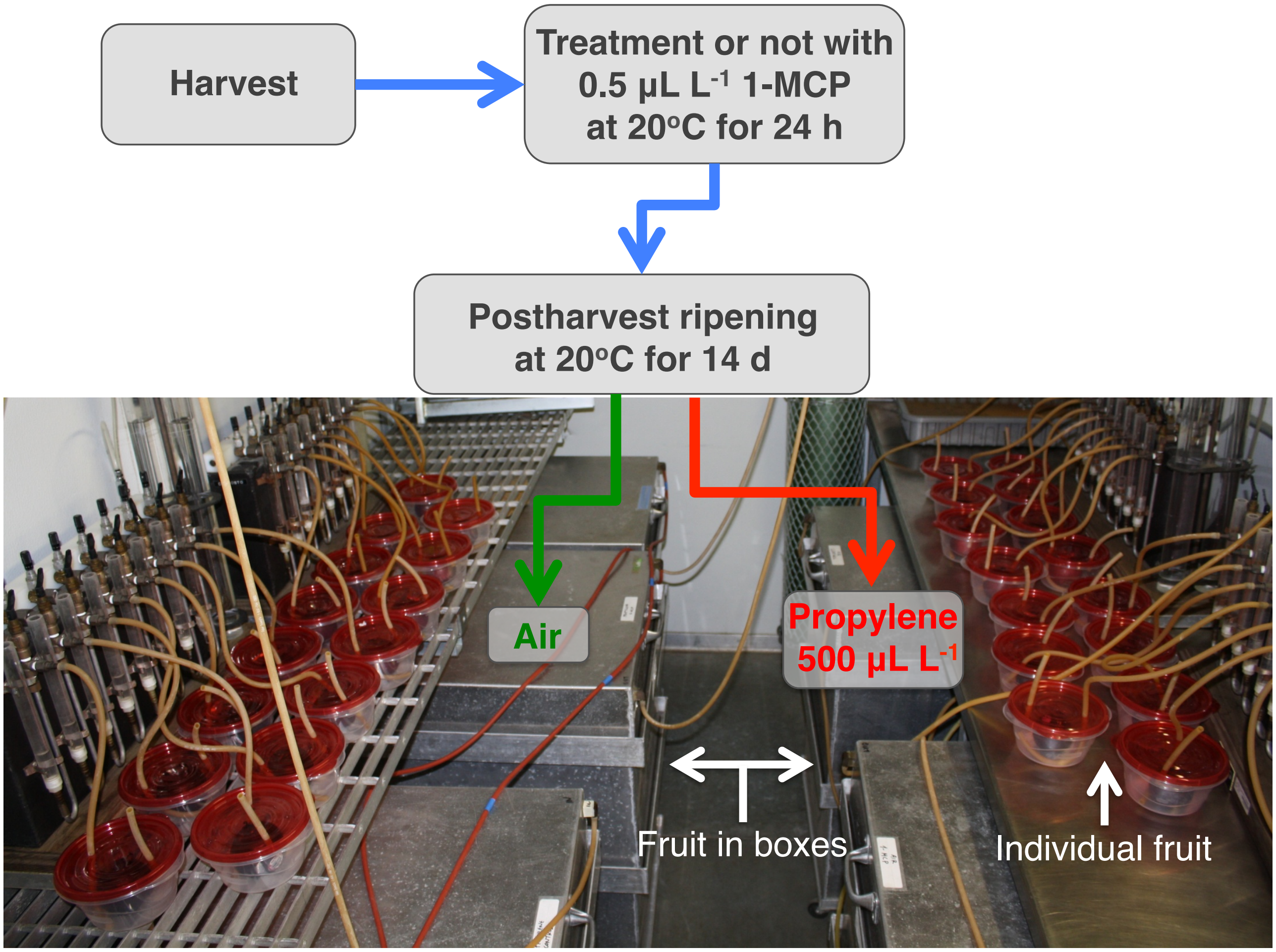

Supplementary Figure 1 | Experimental layout. A climacteric cultivar ("Joanna Red") and two slow-softening cultivars ("Angeleno" and "Sweet Miriam") were harvested and divided into two postharvest treatments: (1) untreated (control, C); and (2) treated with 0.5 µL L^{-1}1-MCP at 20°C for 24 h (1-MCP treatment, M), as previously described (Minas et al., 2013). Immediately after treatment, fruits transferred at room temperature (20°C, 90% RH) to ripen after harvest. During ripening at 20°C, control and 1-MCP-treated fruit were split into two ripening treatments and ventilated continuously with either (1) humidified, ethylene-free air at a flow rate of 2 L min^{-1} or (2) humidified ethylene-free air containing propylene, an analog of ethylene, at a concentration of 500 µL L^{-1} at the same flow rate. Propylene was used to simulate the effect of exogenous ethylene on fruit ripening while allowing the determination of endogenous ethylene production by the fruit during treatment. Ethylene, CO_2 and propylene concentrations were monitored daily. Fruit flesh firmness, soluble solids concentration (SSC), titratable acidity (TA) and skin and flesh color analyzed during ripening at 20°C after harvest at 0 d and every 2 d up to 14 d, as previously described (Minas et al., 2013).

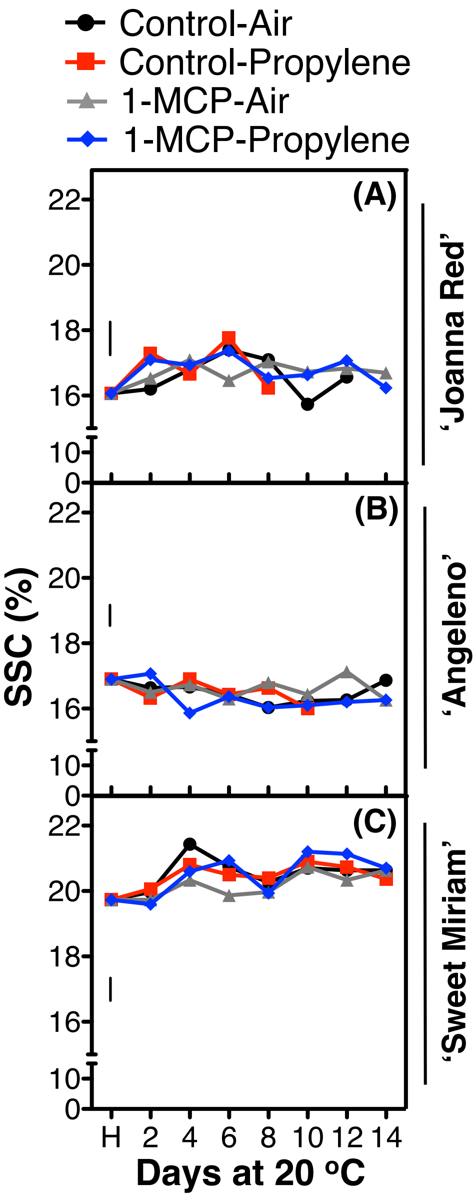

Supplementary Figure 2 | Soluble solids concentration (SSC) changes during postharvest ripening. Changes of SSC during ripening (20°C) under air or propylene (500 µL L^{-1}), immediately after harvest of "Joanna Red" (A), "Angeleno" (B), and "Sweet Miriam" (C) plums previously treated or not with 1-MCP (0.5 µL L^{-1}, 24 h, 20°C). The vertical bars in each particular figure represent the least significant difference (LSD, $P = 0.05$).

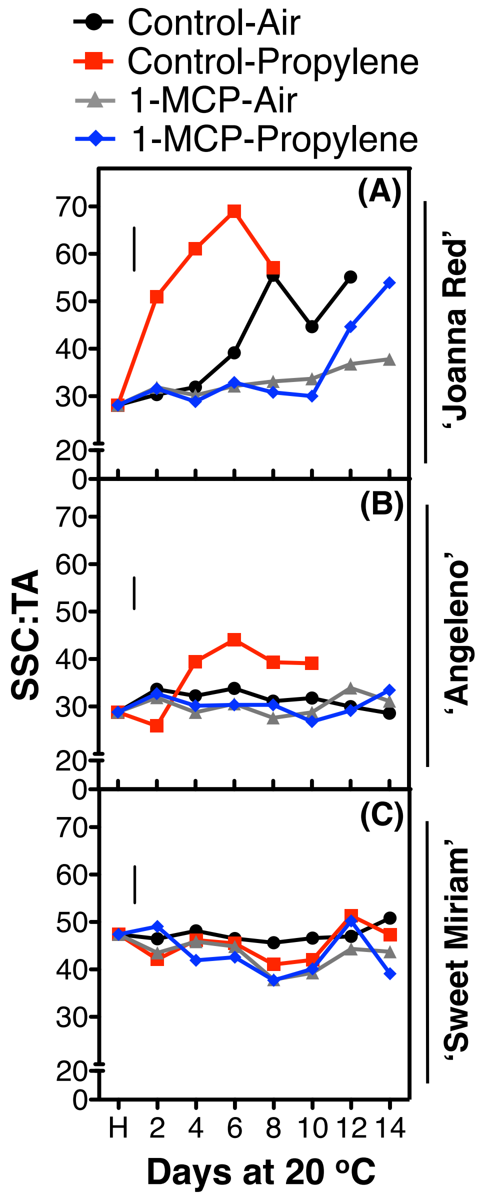

Supplementary Figure 3 | Soluble solids concentration:Titratable Acidity ratio (SSC:TA) changes during postharvest ripening. Changes of SSC:TA during ripening (20°C) under air or propylene (500 μL L^{-1}), immediately after harvest of "Joanna Red" (A), "Angeleno" (B), and "Sweet Miriam" (C) plums previously treated or not with 1-MCP (0.5 μL L^{-1}, 24 h, 20°C). The vertical bars in each particular figure represent the least significant difference (LSD, $P = 0.05$). Abbreviations: SSC: soluble solids content, TA: titratable acidity.

2. Reference

Minas, I. S., Crisosto, G. M., Holcroft, D., Vasilakakis, M., and Crisosto, C. H. (2013). Postharvest handling of plums (*Prunus salicina* Lindl.) at 10°C to save energy and preserve fruit quality using an innovative application system of 1-MCP. *Postharvest Biol. Technol.* 76, 1–9. doi:10.1016/j.postharvbio.2012.08.013.
